# Assessment of Colostrum Quality in Cattle Using Viscosity Measurements

**DOI:** 10.3390/vetsci13060576

**Published:** 2026-06-12

**Authors:** Florian Schneider, Theresa Conze, Kathrin Büttner, Axel Wehrend

**Affiliations:** 1 Veterinary Clinic for Reproductive Medicine and Neonatology, Faculty of Veterinary Medicine, Justus-Liebig University, Frankfurter Strasse 106, 35392 Giessen, Germany; theresaconze@gmail.com (T.C.); axel.wehrend@vetmed.uni-giessen.de (A.W.); 2 Unit of Biomathematics and Data Processing, Faculty of Veterinary Medicine, Justus-Liebig University, Frankfurter Strasse 95, 35392 Giessen, Germany; kathrin.buettner@vetmed.uni-giessen.de

**Keywords:** cattle, colostrum, viscosity, outflow funnel

## Abstract

The provision of high-quality colostrum immediately after birth is essential to ensure adequate passive transfer of immunity in neonatal calves. This study evaluated colostrum viscosity as a potential indicator of colostrum quality, its relationship with immunoglobulin G (IgG) concentration, and the influence of cryopreservation on viscosity measurements. Viscosity was determined in 200 colostrum samples using a cone–plate viscometer and an outflow funnel. IgG concentrations were measured using an enzyme-linked immunosorbent assay (ELISA). Viscosity measured by the plate–cone viscometer averaged 19.33 ± 22.98 cP. No significant differences were observed between viscosity measurements obtained before freezing and after thawing. Flow times through the outflow funnel averaged 18.48 ± 4.27 s. The mean IgG concentration determined by ELISA was 63.89 ± 23.50 mg/mL. Significant positive correlations were identified between viscosity and IgG concentration, between flow time and IgG concentration, and between viscosity and flow time. ROC analysis of the funnel method yielded an AUC of 0.821 (95% CI: 0.752–0.890), indicating good diagnostic performance for identifying poor-quality colostrum (IgG < 50 mg/mL). These findings indicate that viscosity using either a cone–plate viscometer or an outflow funnel is a useful proxy for IgG concentration and may serve as a practical method for assessing colostrum quality. Furthermore, cryopreservation had no detectable effect on colostrum viscosity.

## Simple Summary

The provision of high-quality colostrum immediately after birth is essential for successful passive transfer of immunity in calves. Reliable and practical methods for assessing colostrum quality are therefore of high clinical and economic relevance in cattle production. This study evaluated colostrum viscosity as a potential indicator of colostrum quality and investigated its relationship with immunoglobulin G (IgG) concentration. Two hundred colostrum samples were analysed using a cone–plate viscometer and a standardized outflow funnel, while immunoglobulin concentrations were determined using a laboratory-based immunological assay. In addition, the effect of freezing and thawing on colostrum viscosity was assessed. The results showed no significant differences in viscosity before freezing and after thawing, indicating that cryopreservation does not affect this parameter. Significant positive correlations were found between viscosity using a cone–plate viscometer and IgG concentration, as well as between flow time and IgG concentration. Furthermore, viscosity and flow time were strongly correlated. A receiver operating characteristic (ROC) analysis was conducted to assess the diagnostic performance of the funnel method for classifying colostrum quality. These findings suggest that viscosity measurements obtained using either a cone–plate viscometer or an outflow funnel provide a robust and reproducible indicator of colostrum quality. Measuring viscosity may therefore provide a practical and cost-effective tool for both research and on-farm colostrum assessment.

## 1. Introduction

Adequate colostrum intake is essential for calf health and survival. Due to the synepitheliochorial structure of the bovine placenta, maternal immunoglobulins cannot be transferred effectively to the fetus during gestation. Consequently, passive immunity depends entirely on the timely ingestion of sufficient quantities of high-quality colostrum after birth. In addition to the volume administered, the timing of feeding and the feeding method, colostrum quality is a critical determinant of successful passive transfer [1].

Several methods are available for evaluating colostrum quality. The most accurate approaches are direct measurements of immunoglobulin G (IgG) concentration using enzyme-linked immunosorbent assay (ELISA) or radial immunodiffusion (RID) [2,3,4]. However, these laboratory-based techniques are time-consuming and unsuitable for routine on-farm use, where rapid assessment is required. Consequently, indirect methods such as density measurement and refractive index measurements have been developed and are widely used in practice [5,6].

Despite the availability of these methods, studies have shown that colostrum quality is still insufficiently assessed on many farms [7,8]. Therefore, additional simple and practical methods that can be applied directly under field conditions are needed. Colostrum viscosity has received comparatively little scientific attention as a quality parameter, although many farmers and veterinarians use it subjectively when evaluating colostrum quality [9]. Initial studies reported positive associations between viscosity, assessed using an outflow funnel, and IgG concentration in both bovine and equine colostrum [10,11]. In contrast, another study failed to demonstrate a strong relationship between viscosity and IgG concentration in cattle [12]. Furthermore, viscosity measurements have been used to evaluate the suitability of bovine colostrum for pasteurization [13]. Overall, the available evidence remains limited and inconclusive.

Direct viscosity measurements obtained using a cone–plate viscometer represent an established and widely accepted method in rheological research [14,15,16]. This technique is considered the gold standard for measuring the viscosity of biological fluids and liquid food products [14,17]. Previous studies have applied cone–plate viscometry to biological samples such as blood and sputum [15,16], as well as to dairy products [18]. Moreover, a recent study demonstrated the potential value of viscosity measurements for estimating colostrum quality in mares [11].

Because scientific investigations are often not performed immediately after colostrum collection, it is important to determine whether cryopreservation influences viscosity measurements. Therefore, the present study aimed to evaluate the suitability of direct viscosity measurements obtained with a Brookfield DV2T-LV cone–plate viscometer and to compare them with a simple outflow funnel method, which provides an indirect assessment of viscosity. In addition, the relationships between viscosity, flow time, and IgG concentration were investigated, and the influence of cryopreservation on colostrum viscosity was assessed.

## 2. Materials and Methods

A total of 200 colostrum samples were collected from cattle presented to the clinic as inpatients or outpatients for obstetric care or calving monitoring. The following data were collected for the individual samples:

• Sample number;
• Date of collection;
• Name of the animal owner;
• Ear tag number of the animal;
• Breed;
• Parity;
• Total volume of colostrum.

Colostrum was collected within two hours of parturition. Prior to sampling, gross contamination was removed from the teats, which were subsequently disinfected using cellulose wipes moistened with Spitacid (Ecolab, Monheim am Rhein, Germany). Samples were obtained by manual milking into a teat cup, and the first streams of colostrum were discarded.

Quarters showing visible abnormalities in colostrum appearance were excluded from sampling. A pooled sample was obtained from all clinically normal quarters.

Viscosity measurements using the Brookfield DV2T-LV cone–plate viscometer (Brookfield, Middleboro, MA, USA) and the outflow funnel were performed immediately after sample collection. Remaining colostrum was frozen and stored at −18 °C until further analysis. To evaluate whether viscosity measurements could also be performed after cryopreservation, 29 samples were re-examined following thawing. In addition, thawed colostrum samples obtained from seven dairy farms during routine veterinary colostrum monitoring were included in the analysis.

### 2.1. Viscosity Measurements Using the Brookfield DV2T-LV Cone–Plate Viscometer

Viscosity was measured using a Brookfield DV2T-LV viscometer (Brookfield, Middleboro, MA, USA). The instrument consisted of a device mounted on a stand, a cone screwed to the device, and a sample chamber attached to the device using a cotter pin system. In addition, a circulation temperature control unit (Minichiller 300-H OLÉ, Huber, Offenburg, Germany) was connected to the sample chamber, allowing the sample temperature to be regulated. A shear rate of 3 rpm was used to ensure comparability of the viscosity measurement results. All measurements were carried out using a CPA-40Z measuring spindle (Brookfield Company, Middleboro, MA, USA), which was calibrated to a sample volume of 0.5 mL. Prior to measurement, samples were equilibrated to 30 °C in a water bath (Memmert, Schwabach, Germany). Subsequently, 0.5 mL of colostrum was transferred to the sample chamber using a pipette. Measurements were initiated immediately after loading, and the mean viscosity value recorded after 60 s was documented using RheoCalcT 2.x software and exported for statistical analysis.

### 2.2. Repeatability of the Viscosity Measurement Using the Brookfield DV2T-LV Cone–Plate Viscometer

Thirty bovine colostrum samples were used to assess measurement repeatability. After each measurement, the sample chamber was cleaned and the same sample was reanalysed. This procedure was repeated four additional times, resulting in a total of five viscosity measurements per sample.

### 2.3. Influence of Cryopreservation on Colostrum Viscosity

Twenty-nine bovine colostrum samples were analysed immediately after collection. Following viscosity measurement, samples were stored at −18 °C for two days. Subsequently, samples were thawed in a temperature-controlled water bath at 30 °C and reanalysed once the target temperature had been reached.

### 2.4. Viscosity Measurement Using an Outflow Funnel

A custom-made polypropylene outflow funnel with a capacity of 50 mL and an opening angle of 90° was used for 200 samples. The outlet nozzle had a diameter of 3 mm and a length of 7 mm. Measurements were performed according to the following protocol:

• Samples equilibrated to 30 °C were poured into the funnel until overflow occurred, resulting in a standardized volume of 50 mL.
• The outlet nozzle was closed manually while the funnel was filled.
• The funnel was positioned on a dedicated stand.
• Timing was initiated when the outlet opening was released.
• The measurement was terminated when the first interruption in the liquid column was observed, and the elapsed time was recorded.

### 2.5. Determination of IgG Concentration

IgG concentrations were determined by ELISA at the Institute for Animal Welfare, Behavioural Science, Animal Hygiene and Animal Husbandry, Faculty of Veterinary Medicine, Ludwig Maximilian University of Munich. For analysis, 10 mL of colostrum was transferred to labelled sample tubes, frozen, and transported under frozen conditions to the laboratory. IgG concentrations were measured using a sandwich ELISA according to Erhard et al. [19]. The detailed methodology has been described previously by Sutter et al. [4] and Röder et al. [12]. Samples were diluted 1:50,000 in PBS-Tween and analysed using anti-bovine IgG antibodies. Briefly, 96-well plates were coated with rabbit anti-bovine IgG (5 μg/mL) and blocked with 0.5% gelatin–PBS. Colostrum samples were applied in a two-fold serial dilution series, followed by incubation with peroxidase-conjugated rabbit anti-bovine IgG. After addition of tetramethylbenzidine substrate, the resulting color reaction was measured photometrically and used to calculate IgG concentrations. An IgG concentration of ≥50 mg/mL was used as the threshold for defining good-quality colostrum, as this cutoff is widely accepted in the literature [4].

### 2.6. Statistical Evaluation

The data were recorded using Microsoft Excel (Microsoft Office Excel 2010, Microsoft Corporation, Redmond, WA, USA). Graphical illustrations were generated using GraphPad Prism version 6.07 (GraphPad Software, Inc., La Jolla, CA, USA) and Microsoft Excel (Microsoft Office Excel 2010, Microsoft Corporation Redmond, WA, USA). Descriptive statistics for means and standard deviations were calculated (SAS^®^9.4) [20].

The datasets were non-normally distributed and were analysed using non-parametric statistical methods.

Repeatability of viscosity measurements was assessed using Kendall’s coefficient of concordance (Kendall’s W). To evaluate the effect of cryopreservation, viscosity measurements obtained before freezing and after thawing were compared using a Wilcoxon signed-rank test. In addition, the association between both measurements was assessed using Spearman’s rank correlation analysis.

Furthermore, Spearman’s rank correlation analysis was used to assess the relationships between:

• Flow time and viscosity measured using the cone–plate viscometer;
• Viscosity and IgG concentration;
• Flow time and IgG concentration.

To evaluate the diagnostic performance of the funnel method for the identification of poor-quality colostrum, a receiver operating characteristic (ROC) analysis was performed. Colostrum samples with an IgG concentration below 50 mg/mL, as determined by ELISA, were classified as poor quality. The funnel volume (mL) was used as the test variable, and the IgG-based classification served as the reference standard. The area under the ROC curve (AUC) was calculated to assess the discriminatory ability of the funnel method. The optimal cut-off value was determined using the Youden index. Sensitivity, specificity, positive predictive value (PPV), and negative predictive value (NPV) were calculated for the selected cut-off.

## 3. Results

Colostrum samples were obtained from 200 cows representing 10 different breeds (Table 1). Of these animals, 176 were dairy cattle and 24 were beef cattle. Parity ranged from 1 to 10 lactations, with a mean parity of 3.02. The volume of colostrum obtained at first milking ranged from 200 mL to 15,000 mL, with a mean volume of 4151 mL. A total of 122 samples were analysed immediately after collection, whereas 148 samples were analysed after thawing following cryopreservation.

### 3.1. Viscosity Measured Using the Brookfield DV2T-LV Cone–Plate Viscometer

**Figure 1:**
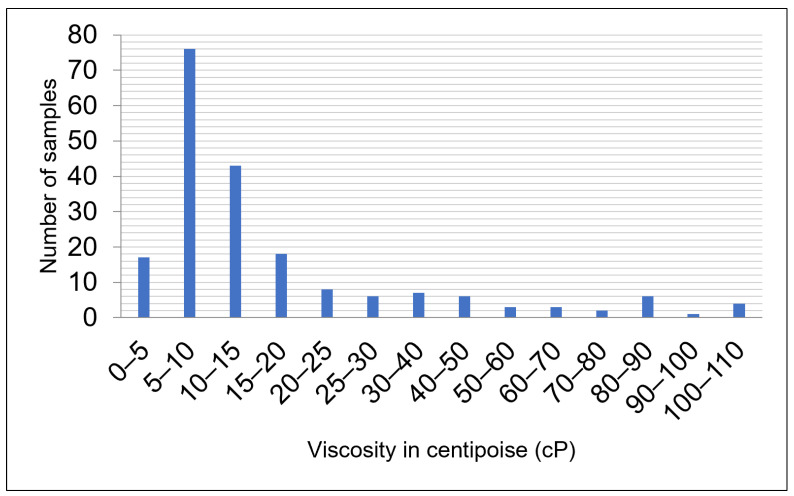
Distribution of bovine colostrum samples according to viscosity measured using a cone–plate viscometer (*n* = 200). Four samples exceeded the viscometer’s measurement range of 102.2 cP and were assigned a value of 110 cP.

Viscosity ranged from 2.25 cP to 110.00 cP. Four samples exceeding the viscometer’s measurement range of 102.2 cP were assigned a value of 110 cP to allow their inclusion in the statistical analysis. The mean viscosity was 19.33 cP ± 22.98 cP. Samples with viscosities below 30 cP predominated (*n* = 168), whereas only a few samples (*n* = 32) had viscosities over 30 cP (Figure 1).

### 3.2. Repeatability of the Viscosity Measurement Using the Brookfield DV2T-LV Cone–Plate Viscometer

Thirty colostrum samples were included in the repeatability analysis. One sample exceeded the measurable range of the viscometer and was excluded from further evaluation. Viscosity values for the remaining samples ranged from 2.25 to 102.1 cP. Kendall’s coefficient of concordance (W) was 0.999 (95% confidence interval), indicating excellent agreement among repeated measurements. The corresponding linear transformation yielded a correlation coefficient of r = 0.998, confirming an exceptionally high level of measurement repeatability.

### 3.3. Influence of Cryopreservation on Colostrum Viscosity

**Figure 2:**
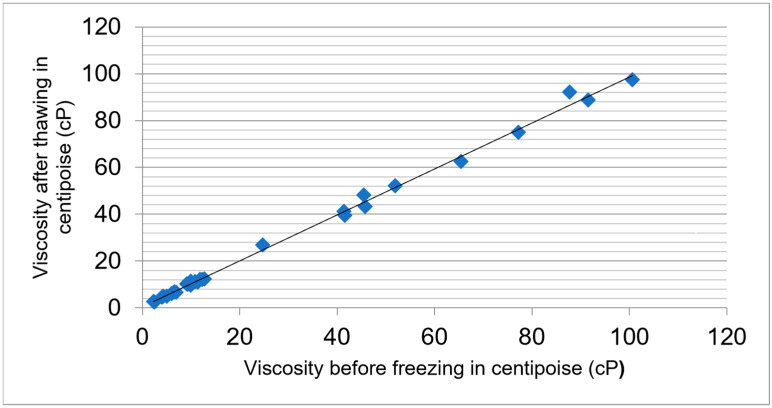
Correlation between viscosity measurements obtained before freezing and after thawing (*n* = 29, ρ = 0.990, *p* < 0.001).

Twenty-nine colostrum samples were included in the analysis after exclusion of one sample that exceeded the measurable range of the viscometer. Mean viscosity was 28.22 cP before freezing and 28.19 cP after thawing. No significant difference was observed between fresh and thawed samples (Wilcoxon signed-rank test: W = 201, *p* = 0.721), indicating that cryopreservation did not affect viscosity measurement. Spearman’s rank correlation analysis further demonstrated a very strong relationship between viscosity measurements obtained before freezing and after thawing (ρ = 0.990, *p* < 0.001; Figure 2).

### 3.4. Viscosity Assessment Using the Outflow Funnel

Flow times ranged from 14.1 to 42.1 s, with a mean value of 18.48 ± 4.27 s. Most samples (*n* = 171) exhibited flow times below 20 s. Samples with longer flow times were less common and displayed greater variability, resulting in a left-skewed distribution (Table 2).

### 3.5. IgG Concentrations

Measured IgG concentrations ranged from 6.6 to 130.0 mg/mL. The mean IgG concentration was 63.89 ± 23.50 mg/mL. Based on the predefined threshold of 50 mg/mL, 61 samples were classified as poor-quality colostrum, whereas 139 samples met the criteria for good-quality colostrum.

### 3.6. Relationship Between Viscosity Measured Using the Plate–Cone Viscometer and Flow Time Using the Outflow Funnel

**Figure 3:**
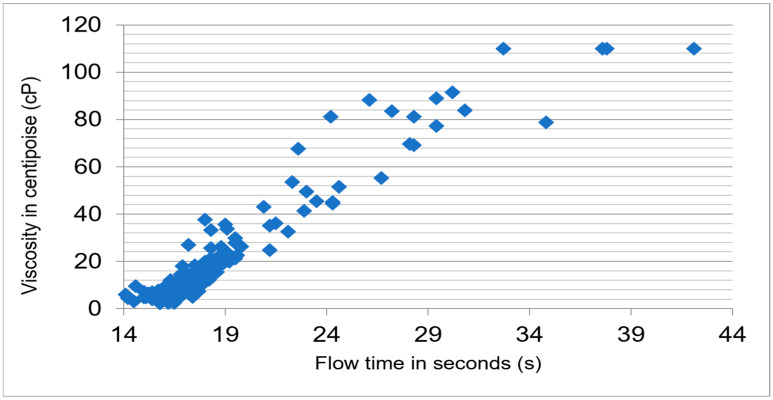
Scatter diagram between flow time measured using the outflow funnel and viscosity measured using a plate–cone viscometer (*n* = 200, ρ = 0.84, *p* < 0.001).

Neither viscosity nor flow time data were normally distributed. Therefore, the relationship between both variables was assessed using Spearman’s rank correlation analysis. A strong positive correlation was observed between viscosity measured with the cone–plate viscometer and flow time measured with the funnel (ρ = 0.84, *p* < 0.001; Figure 3).

### 3.7. Correlation Between Viscosity and IgG Concentration

**Figure 4:**
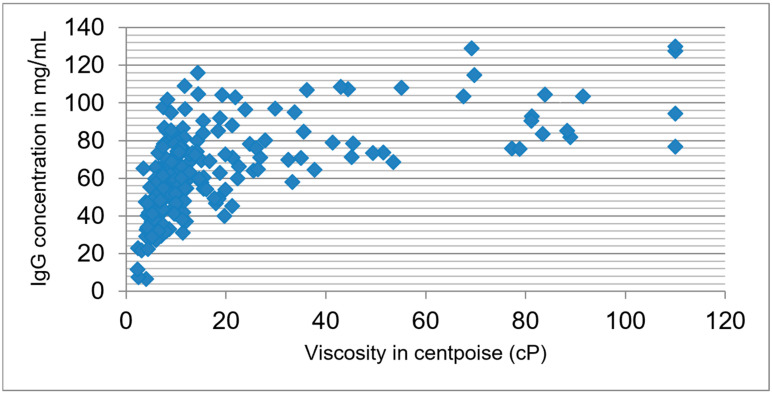
Relationship between viscosity measured using a plate–cone viscometer and the IgG concentration (*n* = 200, ρ = 0.68, *p* < 0.001).

A significant positive correlation was identified between viscosity measured using the cone–plate viscometer and IgG concentration (ρ = 0.68, *p* < 0.001; Figure 4).

### 3.8. Correlation Between Flow Time and the IgG Concentration

**Figure 5:**
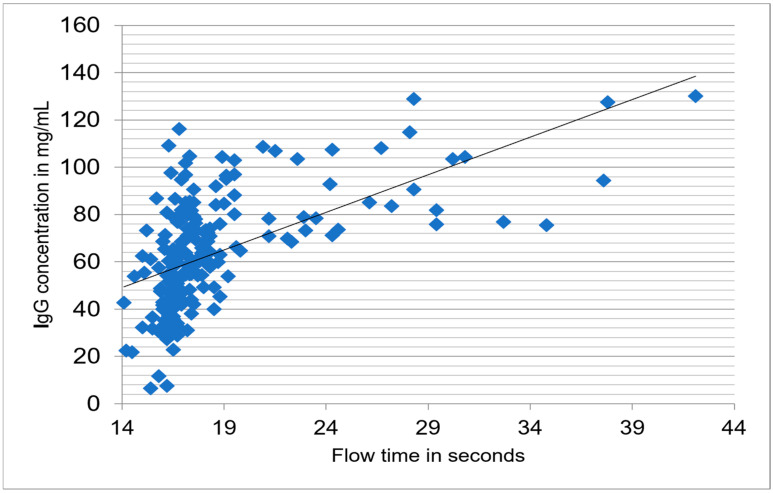
Relationship between flow time measured using the outflow funnel and IgG concentration (*n* = 200, ρ = 0.63, *p* < 0.001).

A significant correlation was observed. The Spearman correlation coefficient (ρ) was 0.63 (*p* < 0.001) (Figure 5).

### 3.9. Receiver Operating Characteristic (ROC) for the Outflow Funnel

Receiver operating characteristic (ROC) analysis was performed to evaluate the ability of the 50 mL funnel method to identify poor-quality colostrum (IgG < 50 mg/mL). The area under the curve (AUC) was 0.821 (95% CI: 0.752–0.890), indicating good diagnostic performance. The optimal cut-off value, determined using the Youden index, was ≤16.9 mL, resulting in a sensitivity of 85.2% and a specificity of 69.8%. At this threshold, the positive predictive value (PPV) was 55.3%, while the negative predictive value (NPV) was 91.5%.

## 4. Discussion

To prevent inadequate passive transfer of colostral antibodies, an early and adequate supply of high-quality colostrum must be ensured in newborn calves [1]. Colostrum quality is primarily determined by its immunoglobulin G (IgG) concentration, among other factors.

In the present study, considerable variation in colostral IgG concentrations was observed. Such variation may result from both animal-related and environmental factors. Conneely et al. [21] investigated factors affecting colostrum quality and identified farm management and season as important environmental influences. Among animal-related factors, parity, the interval between calving and first milking, and first-milking volume significantly affected colostrum quality. Similarly, Zentrich et al. [22] reported a negative effect of humidity and a positive effect of dry period length on colostrum quality. As samples in the present study were collected throughout an entire year, part of the observed variation in IgG concentrations may be attributed to seasonal influences.

In contrast to previous studies that focused exclusively on Holstein cows [6,23], the present study included colostrum samples from ten different cattle breeds. However, one limitation of this study is the unequal breed distribution within the study population. The majority of cows were Simmental cattle (162/200), whereas only 47 Holstein Friesian cows were included. This imbalance reflects the regional dairy farming structure, as most samples were collected in an area where Simmental cattle are the predominant dairy breed. Consequently, the findings may not be fully representative of dairy populations with different breed compositions. Future studies should include a more balanced breed distribution to enable direct comparisons between Simmental and Holstein Friesian cows and to investigate potential breed-related differences in colostrum quality and funnel test performance.

Because cows originated from different farms, variation in management practices, including dry period length, feeding strategies, and the interval between calving and milking, may also have contributed to the observed differences in IgG concentrations. Since the primary objective of this study was to investigate the relationship between IgG concentration and viscosity, a broad range of IgG concentrations was intentionally included.

Direct measurement of IgG concentration in colostrum is limited to laboratory-based methods such as enzyme-linked immunosorbent assay (ELISA) and radial immunodiffusion (RID), which restrict their use under field conditions. Therefore, several indirect methods for assessing colostrum quality, including density and refractive index measurements, have become established in cattle [5,6,24]. Because IgG concentration was used as the reference method in the present study, these indirect assessment methods were not evaluated. The use of an outflow funnel for estimating bovine colostrum quality has previously been investigated in only two studies [10,12], with conflicting results.

For mare colostrum, the relationship between viscosity and IgG concentration has already been demonstrated [11]. In cattle, direct viscosity measurements have been reported only once, in a study investigating the suitability of colostrum for pasteurization using a limited number of samples [13]. In the present study, viscosity was measured using a cone–plate viscometer, which is considered the gold standard for viscosity determination.

Direct measurement of the viscosity of bovine colostrum has so far only been carried out in one study to assess the suitability for pasteurization in a small number of samples [13]. Viscosity was directly measured using a plate–cone viscometer, which is considered the gold standard for viscosity measurement. The implementation and operation of this device are simple.

Repeatability of viscosity measurements was assessed using Kendall’s W. The resulting coefficient (r = 0.998) indicated excellent agreement between repeated measurements. Previous studies evaluating the repeatability of cone–plate viscometers for biological fluids such as blood and oil reported deviations of only 2–3% [25,26], which is consistent with the findings of the present study. To evaluate the effect of cryopreservation, viscosity measurements were performed before freezing and after thawing. No significant differences were observed. This finding is consistent with previous studies on milk and dairy products, which reported no significant effect of freezing on viscosity [27,28,29]. After confirming that cryopreservation did not affect viscosity, frozen colostrum samples were used in the main experiment.

A correlation was observed between colostrum viscosity and IgG concentration. In contrast, Hallberg et al. [30] and Maunsell et al. [31] did not identify such a relationship. However, in both studies, viscosity was assessed subjectively by classifying samples into viscosity categories rather than by objective measurement techniques.

As a cone–plate viscometer is not suitable for measuring viscosity in practice, the flow time of the colostrum from a defined outflow funnel was tested as an indirect parameter of viscosity. For this purpose, a Spearman rank correlation analysis was calculated between the outflow funnel flow time and the viscosity measured using a plate–cone viscometer, which was 0.84 (*p* < 0.001). This means that the measurement of flow time is suitable for estimating viscosity under practical conditions. In addition, a significant correlation was found between flow time and the IgG content of the colostrum. This confirmed the correlation between outflow funnel flow time and IgG content described by Kritzinger [10].

Kritzinger [10] carried out a receiver operating characteristic (ROC) analysis to determine the optimal cut-off value for distinguishing between good- and poor-quality colostrum using an outflow funnel. A flow time of 23.5 s measured with a 100 mL outflow funnel was identified as the optimal threshold, resulting in a sensitivity of 77.8% and a specificity of 73.9%. Röder et al. [12] were less convinced of the usefulness of the outflow funnel method. Using ROC analysis and the same cut-off value, they reported a sensitivity of 65.2% and a specificity of 76.1%. In the present study, ROC analysis of the 50 mL outflow funnel yielded an area under the curve (AUC) of 0.821 (95% CI: 0.752–0.890), indicating good diagnostic performance. The optimal cut-off value determined by the Youden index was ≤16.9 mL, resulting in a sensitivity of 85.2% and a specificity of 69.8%. Furthermore, the positive predictive value was 55.3%, while the negative predictive value reached 91.5%, suggesting that the method is particularly suitable for ruling out poor-quality colostrum.

Several methodological differences may explain the discrepancies between studies. Röder et al. [12] emphasized that a sample temperature of 30 °C is required to obtain optimal results. However, although sample temperatures were measured using a digital thermometer, the samples were not standardized to 30 °C prior to analysis and ranged from 27.3 ± 9.4 °C to 38.2 ± 2.6 °C. In contrast, all samples in the present study were measured at a standardized temperature of 30 °C. This is particularly relevant because viscosity is strongly temperature-dependent and therefore directly affects flow-based measurements.

Currently Brix refractometry the most widely used on-farm method for assessing colostrum quality because it is rapid, inexpensive, and easy to perform [32,33]. In addition, Brix values have been shown to correlate well with colostral IgG concentrations. However, Brix refractometry does not directly measure immunoglobulins but rather the total dissolved solids in colostrum. Consequently, factors other than IgG may influence the measurement. Sockett et al. (2023) [32] reported a strong correlation between Brix values and IgG concentrations but concluded that Brix refractometry is better suited for categorizing colostrum quality than for accurately predicting exact IgG concentrations. Similar to Brix refractometry, the funnel method represents an indirect approach for estimating colostrum quality. In the present study, the 50 mL funnel achieved an AUC of 0.821, indicating good diagnostic performance. The high negative predictive value (91.5%) suggests that the method is particularly useful for identifying colostrum that is unlikely to be of poor quality. In contrast, the lower positive predictive value (55.3%) indicates that some samples classified as poor quality by the funnel method would still exceed the IgG threshold of 50 mg/mL. As viscosity is influenced not only by IgG concentration but also by other colostral components such as fat, protein, total solids, and temperature, strict standardization of measurement conditions is required. Nevertheless, the funnel method offers practical advantages, as it requires only simple equipment and can be performed rapidly under field conditions without the need for optical instruments. Overall, both methods should be regarded primarily as screening tools for categorizing colostrum quality rather than as methods for determining exact IgG concentrations. Further studies directly comparing the diagnostic performance of funnel-based viscosity assessment and Brix refractometry under field conditions would be valuable.

## 5. Conclusions

This study demonstrated significant correlations between viscosity (measured by both cone–plate viscometer and outflow funnel) and colostral IgG content. Thus, the outflow funnel is a suitable complementary tool for assessing colostrum quality. The task of future investigations will be to determine the cut-off values for differentiating between good and poor colostrum for different volumes and colostrum temperatures.

## Figures and Tables

**Table 1:** Distribution of cattle by breed and production type in the study population (*n* = 200).

Breed	Number of Animals Sampled	Production Type
Simmental Milk	126	Milk
Holstein Frisian	47	Milk
Simmental Beef	9	Beef
Charolais	6	Beef
Limousin	4	Beef
Crossbreed	4	2 milk, 2 beef
Red Holstein	2	Milk
Red Heights	2	Beef
Hereford	1	Beef
Lineback	1	Milk

**Table 2:** Distribution of bovine colostrum samples according to flow time measured using the 50 mL outflow funnel (*n* = 200).

Flow Time in s	Number of Samples
14.1–19.9	171
20.0–29.9	22
30.0–39.9	6
40.0–42.1	1

## Data Availability

The original contributions presented in this study are included in the article. Further inquiries can be directed to the corresponding author.

## Abbreviations

The following abbreviations are used in this manuscript:

ELISA	Enzyme-linked immunosorbent assay
IgG	Class G immunoglobulins
RID	Radial immunodiffusion
